# Development of an amplified luminescent proximity homogeneous assay for the detection of sulfonamides in animal‐derived products

**DOI:** 10.1002/fsn3.2443

**Published:** 2021-07-12

**Authors:** Yong Jin, Yanping He, Dali Zhao, Yan Chen, Qiang Xue, Mingqiang Zou, Hong Yin, Shige Xing

**Affiliations:** ^1^ Chinese Academy of Inspection and Quarantine Beijing China; ^2^ Anhui Normal University Wuhu China; ^3^ Jilin International Travel Health Care Center (Changchun Customs Port Clinic) Changchun China

**Keywords:** amplified luminescent proximity homogeneous assay (AlphaLISA), food quality control, sulfamethazine, sulfonamides

## Abstract

In this study, we carried out an amplified luminescent proximity homogeneous assay (AlphaLISA) to detect sulfonamides (SAs) antibiotic residues in plasma, milk, pork, chicken, and fish. The SAs AlphaLISA method can detect 13 SAs with half‐inhibitory concentration (IC_50_) 2.11–29.77 ng/ml. The detection level of those SAs was 0.3–41.12 ng/ml in matrices, which satisfied the maximum residue limit (MRL) of the European Union, United States, and China. Our recoveries are in the range of 88% to 116.8% with a coefficient of variation less than 9.3% for different spiked food samples. We observed a good correlation between the AlphaLISA and liquid chromatography–tandem mass spectrometry (LC‐MS/MS) with blood samples from injected rabbits. The established AlphaLISA method provided a no‐washing, rapid, high‐throughput screening tool for SAs in food quality control, which is suitable for small‐volume samples.

## Graphical abstract

An amplified luminescent proximity homogeneous assay (AlphaLISA) was carried out in the detection of 13 SAs antibiotic residues in foods with the half‐inhibitory concentration (IC50) values from 2.11 to 29.77 ng/ml. Good correlations were observed between the AlphaLISA and liquid chromatography–tandem mass spectrometry (LC‐MS/MS) results for blood samples from injected rabbits.

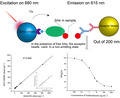

## 1 INTRODUCTION

Sulfonamides (SAs) are a group of synthetic chemotherapeutics with a common *p*‐aminobenzene sulfonamide moiety. They have been widely used as veterinary antibiotic drugs to prevent bacterial infectious diseases and promote animal husbandry and fish farming since last century (Liu et al., 2016). The widespread use of SAs in animal husbandry, and noncompliance with withdrawal time, can enrich chemical residues in animal‐derived foods. Furthermore, the SAs residues in foods can create potential human health hazards to cause thyroid follicular tumors, allergic reactions, and antibiotic resistance. To ensure food safety for consumers, European Union and China have set the MRL for edible animal products including meat and milk to 100 ng/ml for total SAs content, and the United States has set 100 ng/ml for most SAs in edible animal products (Administration, 2018; Agriculture, 2002; Union, 2010).

Several instrumental analysis techniques have been proposed for the determination of SAs residue (Premarathne et al., 2017; Wang et al., 2019; Xie et al., 2020; Yang et al., 2018; Zhao et al., 2018), including liquid chromatography (LC), liquid chromatography–tandem mass spectrometry (LC‐MS/MS), and ultra‐high‐performance supercritical fluid chromatography (SFC). All these detection techniques have earned robust recognition in SAs and have many advantages, such as large dynamic linear range, low detection limits, and high productivity. However, most of these are complicated, time‐consuming, and costly. Meanwhile, immunoassay methods are being developed rapidly in screening large numbers of sample because of its high throughput, short detection time, reduced sample consumption, and low overall cost (Li, Liang, et al., 2018; Li et al., 2019, 2020; Liang et al., 2019). Enzyme‐linked immunosorbent assay (ELISA) methods are one of the popular immunoassay methods for SA multiresidue screening (Adesiyun, 2020; Krall et al., 2018; Shelver et al., 2008). However, ELISA usually requires many times washing which makes the whole detection time often last for 2–3 hr and cannot meet the need of rapid detection (Yu et al., 2015).

**FIGURE 1:**
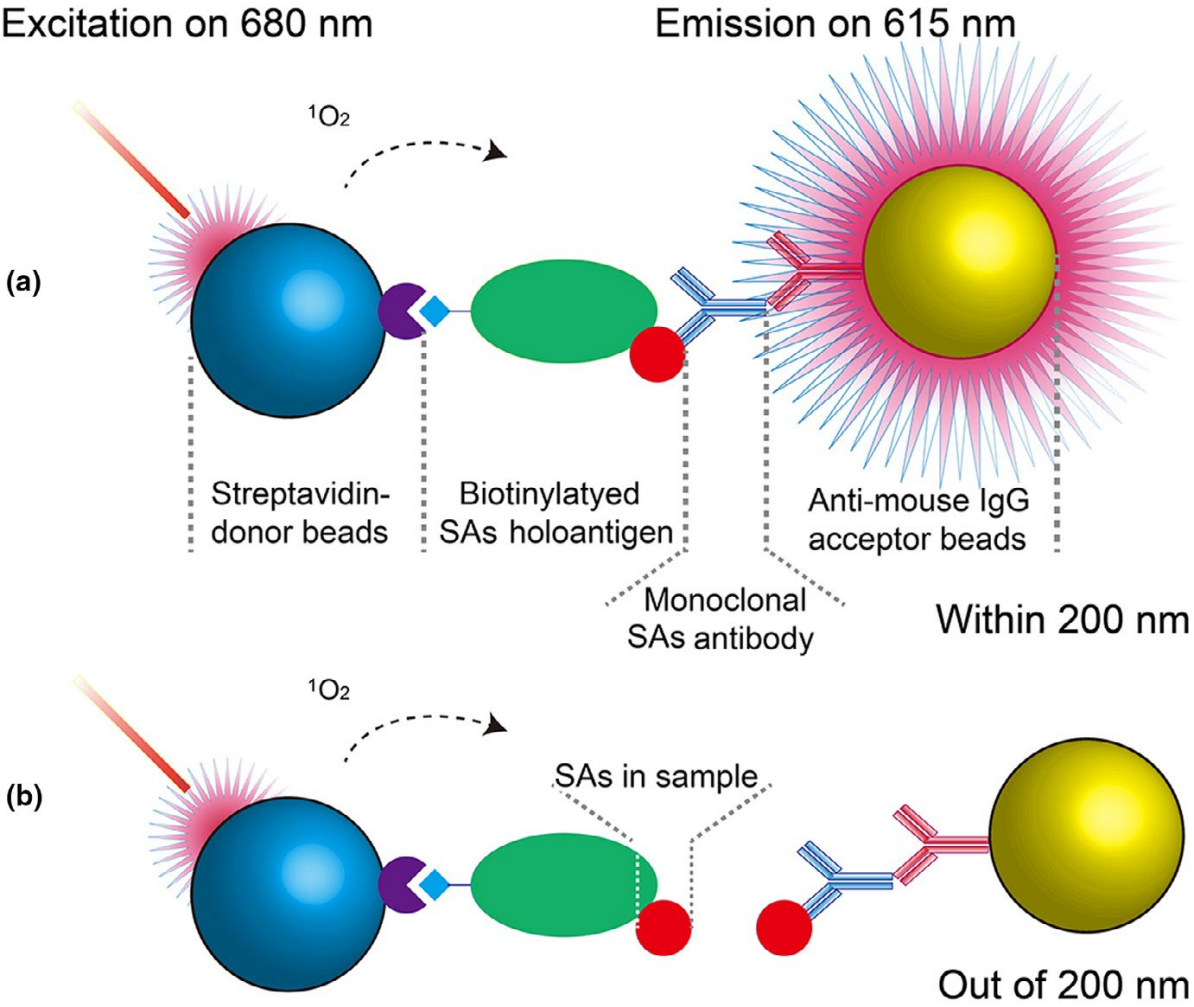
Principle of SAs detection based on AlphaLISA. (a) When there was no free SAs, the acceptor beads were in an emitting state (“bright”) owing to the singlet oxygen (^1^O_2_) generated by donor beads upon photo‐excitation at an effective assay distance of 200 nm; (b) in the presence of free SAs, the acceptor beads were in a nonemitting state (“dark”) because the donor beads were out of the 200 nm range

AlphaLISA technology is a homogenous light‐induced chemiluminescence immunoassay in which donor beads (embedded phthalocyanine) can produce singlet oxygen (^1^O_2_) by photo‐excitation (λ_ex_ = 680 nm); the singlet oxygen then forms acceptor beads (embedded thioxene/EuIII‐chelate mixtures) to generate fluorescence (λ_emi_ = 615 nm) at an effective assay distance of 200 nm owing to the long life span of the singlet oxygen (0.4 μs), with the emission decay of the fluorescence being approximately 200 μs (Figure 1). The AlphaLISA has many advantages over the ELISA; for example, its high sensitivity, relatively quick testing time, reduced hands‐on workflow resulting from the ability to sequentially overlay the reagents, and it is easily adaptable to automation and high‐throughput screening (Bielefeld‐Sevigny, 2009; Li, Chen, et al., 2018; Wang et al., 2020). Therefore, AlphaLISA has been applied for the target detection, kinase assays, and protein–protein interactions (Armstrong et al., 2018; Lassabe et al., 2018; Zhao et al., 2019).

In the present study, we evaluated AlphaLISA system for SAs detection in plasma, milk, pork, chicken, and fish and compared its capability to conventional LC‐MS/MS.

## 2 MATERIALS AND METHODS

### 2.1 Chemicals and instruments

The SAs holoantigen and SAs monoclonal antibody were produced by our laboratory. SA standard substance includes sulfamethazine (SMT), sulfamethoxydiazine (SMD), sulfisomidine (SFM), sulfamerazine (SMR), sulfamonomethoxine (SMM), sulfaquinoxaline (SQX), sulfadimethoxine (SDM), sulfadiazine (SDZ), sulfachloropyridazine (SCP), sulfaclozine (SCL), sulfamethizole (SMZ), sulfamoxole (SXL), sulfamethoxypyridazine (SMP), sulfadoxine (SDOX), sulfathiazole (STA), sulfapyridine (SP), sulfamethoxazole (SMX), sulfisoxazole (SIZ), trimethoprim (TMP), sulfanitran (SNT), sulfaguanidine (SG), sulfaphenazole (SUL), sulfacetamide (SAM), and sulfabenzamide (SBZ) were obtained from Dr. Ehrenstorfer (Augsburg, Germany). Bovine serum albumin was acquired from Equitech‐Bio, Inc. (Kerrville, TX, USA). Sulfo‐NHS‐LC‐Biotin was obtained from Thermo Fisher Scientific Inc. (Waltham, MA, USA). Goat anti‐mouse IgG, Na cyanoborohydride powder (NaBH_3_CN), and carboxymethoxylamine were acquired from Sigma‐Aldrich Co. (St. Louis, Mo, USA). Tween‐20 was obtained from Thermo Fisher Scientific Inc. (Waltham, MA, USA). 4‐(2‐Hydroxyethyl)‐1‐piperazineëthanesulfonic acid (HEPES) (1 M, pH = 7.4) was acquired from Invitrogen (Carlsbad, CA, USA). Amicon® Ultra‐15 centrifugal filters (30 kDa) were purchased from Millipore Ltd. (Billerica, MA, USA). Streptavidin‐coated donor beads, anti‐mouse IgG acceptor beads, white 96‐well microplates, and immunoassay buffer were purchased from PerkinElmer (Boston, MA, USA). Chemicals and reagents including ethyl acetate and *n*‐hexane were analytical grade and were purchased from Sinopharm Chemical Reagent Co., Ltd. (Shanghai, China). SMT sodium injection was obtained from Shennong Animal Pharmaceutical Co. Ltd. (Hunan, China). The rabbits were supplied by Beijing Vital River Laboratory Animal Technology Co., Ltd.

The AlphaLISA reader was supplied by Tianjin University (Tianjin, China). Transmission electron microscopy (Tecnai G^2^ F30, FEI, USA) was used to characterize the acceptor beads in different stages. LC‐MS/MS was supplied by Waters Corporation (Waters ACQUITY UPLC I‐Class XevoTQ‐XS, USA).

Statistical analysis of the data was performed using RIDAWIN software from R‐BioPharm AG (Darmstadt, Germany).

### 2.2 Preparation of antigen and acceptor beads

The SAs holoantigen (10 mg/ml) was prepared in phosphate‐buffered saline (PBS), to which 17.2 µL of 10 mmol/L Sulfo‐NHS‐LC‐Biotin reagent solution was added according to the calculation provided in the reagent instructions. The reaction was then incubated at room temperature for 30 min. Excess nonreacted biotin and reaction by‐products were removed by using a centrifugal filter (30 kDa). The final concentration of biotinylated SAs holoantigen was 1 mg/ml (15 μmol/L).

The acceptor beads were prepared as follows: A mixture (200 μL) of 1 mg naked acceptor beads, 0.1 mg anti‐mouse IgG, 10 μL NaBH_3_CN (400 mmol/L), and 1.25 μL 10% Tween‐20 (100 mmol/L HEPES, pH = 7.4) was incubated at 37°C for 24 hr with mild agitation in dark. Then, 10 μL carboxymethoxylamine was added and incubated at 37°C for 1 hr in dark for block unreacted sites; the block solution was removed by centrifugation (4°C, 13000g, 60 min). Next, 0.5 μL 5 mg/ml goat anti‐mouse IgG acceptor beads was added into 500 μL AlphaLISA buffer; then 0.5 μL 10 mg/ml (63 μmol/L) SAs monoclonal antibody was added; and the mixture was incubated at 37°C for 1 hr with mild agitation in dark. The buffer and excess antibody were removed by centrifugation (4°C, 13,000 g, 60 min). The obtained preparations were store at 4°C.

### 2.3 Characterization of acceptor beads

The dimensional characteristics of acceptor beads were conducted on TEM at an accelerating voltage of 300 keV. For this purpose, the naked acceptor beads and their conjugates with antibodies were dispersed in ultrapure water and applied onto a carbon‐supported 300‐mesh copper specimen grid to dry under a heat lamp prior to the detection.

### 2.4 Procedure of AlphaLISA

In a typical experiment, 15 µl of samples, 10 µl of biotinylated SAs holoantigen, 10 µl of SAs monoclonal antibody, and 15 µl of anti‐mouse IgG‐coated acceptor beads were sequentially added into the microplate and incubated at 37°C in dark, and then, the streptavidin‐coated donor beads were added to each well. After incubation for an additional 15 min at 37°C, the signal was read by AlphaLISA reader.

### 2.5 Assay optimization

The concentrations of SAs holoantigen and SAs monoclonal antibody, and reaction time were examined to improve the sensitivity of the assay. To evaluate the effect of the antigen and antibody, different concentrations of the SAs holoantigen (0–25 nM) and the SAs monoclonal antibody (0–200 nM) were tested in a cross‐over study designed using recommended concentrations of donor beads (17.91 ng/ml) and acceptor beads (5 ng/ml) without free SAs to obtain the best AlphaLISA signal. Then, fixing the concentration of the SAs holoantigen, changing the concentration of the SAs antibody along with that of the free SMT.

The effects of reaction time are also investigated by detecting the AlphaLISA signals of the SMT standard curve at different times from 20 to 70 min. The best SAs calibration curve was used to evaluate both the optimal concentration of antibody and total reaction time.

### 2.6 Sensitivity and specificity of the SA AlphaLISA method

Under optimized conditions, the standard curves were generated by using SAs standard substance that was prepared by diluting appropriate amounts of SAs in PBS solution (0.01 M PBS, pH = 7.4). The sensitivity of this AlphaLISA was evaluated by the half‐inhibitory concentration (IC_50_) values of the SAs standard curves using RIDAWIN software. The limit of detection (LOD) was determined by the mean background levels plus 3 times *SD* (Bo +3 *SD*).

To investigate the specificity of the SAs AlphaLISA method, cross‐reactivity (CR) with SAs (including SMT, SMD, SFM, SMR, SMM, SQX, SDM, SDZ, SCP, SCL, SMZ, SXL, SMP, SDOX, STA, SP, SMX, SIZ, TMP, SNT, SG, SUL, SAM, and SBZ) was calculated. The corresponding CR of each antibiotic in each test was quantitated from the individual standard curve based on the following formula: CR (%) = (IC_50_ of SMT)/(IC_50_ of SAs) × 100%.

The concentration of each sulfonamide (*C*_SA_) was obtained using the following equation: *C*_SA_ = *C*_SMT_ × CR, where *C*_SMT_ is the concentration calculated from the calibration curve as SMT equivalents and CR is the cross‐reactivity.

### 2.7 Preparation of rabbits

The rabbits (2–3 kg) were injected intramuscularly with 10 mg/kg SMT in their hind legs every 24 hr for three consecutive days. Blood was sampled every 1 hr to obtain 10 blood samples from the rabbit ear‐rim vein. In addition, a 10 ml aliquot of blood was collected prior to the SMT injection as a negative control. All of the blood samples were stored in tubes containing antithrombin, impurities were removed by centrifugation, and the samples were frozen at −30°C until analysis. The animal experiment was reviewed and approved by Institutional Animal Care and Use Committee (IACUC) and was conducted in accordance with relevant guidelines and regulations.

### 2.8 Preparation of samples

Milk, pork, chicken, fish, and plasma were used as animal‐derived food sample for SAs residue analysis, and they were verified to be negative samples by LC‐MS/MS. 4 ml or 4 g of samples was spiked with the SMT to three different levels (25, 50, and 100 ng/ml or ng/g), and each level of the sample was analyzed in triplicate.

For the AlphaLISA analysis, the sample was prepared according to the report (Dmitrienko et al., 2014). Basically, 4 g homogenized sample (pork, chicken, or fish) was extracted with 4 ml of ethyl acetate by vortex (10 min). After centrifugation (5 min, 2000 g, 25°C), 1 ml of supernatant was dried under a nitrogen stream (40°C) and then dissolved into 1 ml of PBS. 1 ml of *n*‐hexane was added for defatting with centrifugation (5 min, 2000 g, 25°C); the lower aqueous phase was diluted 20‐fold with PBS before analysis. As for liquid samples, 2.5 ml of skimmed milk or 1 ml of plasma was extracted with 5 ml of ethyl acetate by vortexed for 1 min. After centrifugation (2 min, 2000 g, 25°C), 2 ml of supernatant was dried, dissolved, defatted, and centrifuged as meat samples.

For the LC‐MS/MS analysis, 1 ml sample of plasma (to which had been added 20 µL of phosphoric acid) and 3 ml of acetonitrile (precooled at 4°C for 1 hr) were vortexed for 5 min, followed by cooling at 4°C and centrifugation (10 min, 15,000 g, 25°C). The supernatant was dried under a nitrogen stream (40°C) and then reconstituted into 1 ml of acetonitrile‐water (1:9) with 0.1% formic acid. Then, 1 ml of *n*‐hexane was added for defatting, followed by centrifugation (5 min, 2000 g, 25°C); the lower aqueous phase was filtered through a 0.22‐µm filter prior to analysis.

## 3 RESULTS

### 3.1 Morphology of the acceptor beads

**FIGURE 2:**
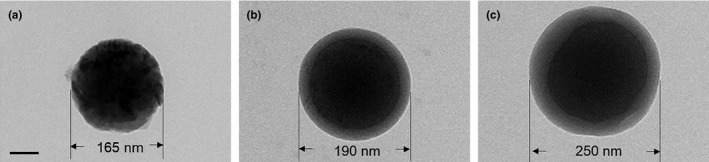
Morphology of acceptor beads as imaged by transmission electron microscopy. (a) Naked acceptor beads; (b) anti‐mouse IgG‐coated acceptor beads; and (c) monoclonal antibody‐bound anti‐mouse IgG‐coated acceptor beads. The scale bar was 50 nm

First, we observed the morphological change of acceptor beads in different stages (Figure 2). The diameter was 165 nm of the naked acceptor beads, which was increased to 190 nm of anti‐mouse IgG‐coated acceptor beads and 250 nm of monoclonal antibody‐bound anti‐mouse IgG‐coated acceptor beads. The increase in diameter of the acceptor beads suggested that the SAs monoclonal antibody was effectively bound to the anti‐mouse IgG‐coated acceptor beads, which also confirmed the principle of SAs AlphaLISA methods (Figure 1).

### 3.2 Optimization of assay conditions

**FIGURE 3:**
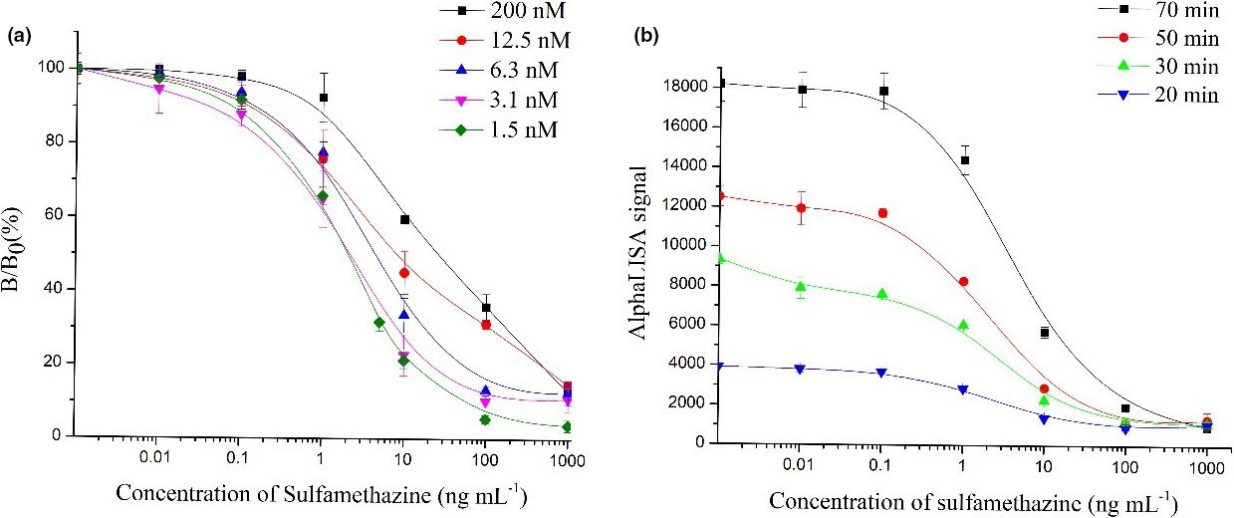
Optimization of concentration of the SAs antibody (a) and total reaction time (b). Note: B/B_0_(%) equals AlphaLISA signal values of standard or sample divided by AlphaLISA signal values at zero standard concentration times 100. The vertical bars indicate standard deviation of the mean results of three replicates. The different lines represent different concentrations of the SAs antibody(a) and total reaction time (b)

Next, we used a two‐step method for optimizing the concentration of antigen and antibody. First, the we obtained best AlphaLISA signal when the concentrations of SAs holoantigen and SAs monoclonal antibody were 12.5 and 200 nM, respectively, in a cross‐over study (Table 1). It looked like the change of readout signals was in a reversed trend compared with other groups when there was no antibody. We considered that was the reasonable fluctuation of electric noise of AlphaLISA reader system. Moreover, these movements have little effect on the detection results and could be generally ignored. Subsequently, fixing the concentration of the SAs holoantigen (12.5 nM), the SMT calibration curve changed along with the different concentrations of the SAs antibody. The IC_50_ values were 2.11, 2.71, 6.58, 14.04, and 33.93 ng/ml when the concentration of SAs antibody was 1.5, 3.1, 6.3, 12.5, and 200 nM, respectively (Figure 3a). Meanwhile, when the concentration of the SAs monoclonal antibody was below 1.5 nM, the AlphaLISA signals became weak and unstable, which was difficult to record. Therefore, 1.5 nM of the SAs monoclonal antibody was chosen as the optimal value. This two‐step method was conducted stepwise, which differed from the strategy used in previous studies and resulted in more convincing optimization values.

**TABLE 1:** Cross‐over study of concentrations of SAs holoantigen and SAs monoclonal antibody

Concentration of SAs monoclonal antibody (nM)	Concentration of SAs holoantigen (nM)					
	25	12.5	6.25	3.13	1.56	0
0	793	809	872	920	956	1,119
12.5	19,372	19,043	13,321	8,074	5,424	957
25	25,731	25,352	18,314	10,923	6,800	879
50	29,874	29,721	23,181	13,746	8,227	791
100	31,886	33,378	26,686	15,962	7,878	672
200	34,148	34,563	29,526	22,061	11,630	617

Note

Readouts in the table represent the average of AlphaLISA signals without free SAs.

Having obtained the optimal concentration of SAs holoantigen and SAs monoclonal antibody, we investigated effects of reaction time, because it takes time for the reaction between the target and antibody and we would like to find optimal time for practical applications. As shown in Figure 3b, the best IC_50_ = 1.53 was achieved when the total reaction time of 30 min. Therefore, 30 min with reaction was selected as the optimal time and procedure for subsequent experiments.

Based on the results from the above optimization steps, we have come up with the following conditions; the concentrations of SAs holoantigen and SAs antibody were 12.5 nM and 1.5 nM; and the total reaction time was 30 min.

### 3.3 Sensitivity and specificity of the SAs AlphaLISA method

**FIGURE 4:**
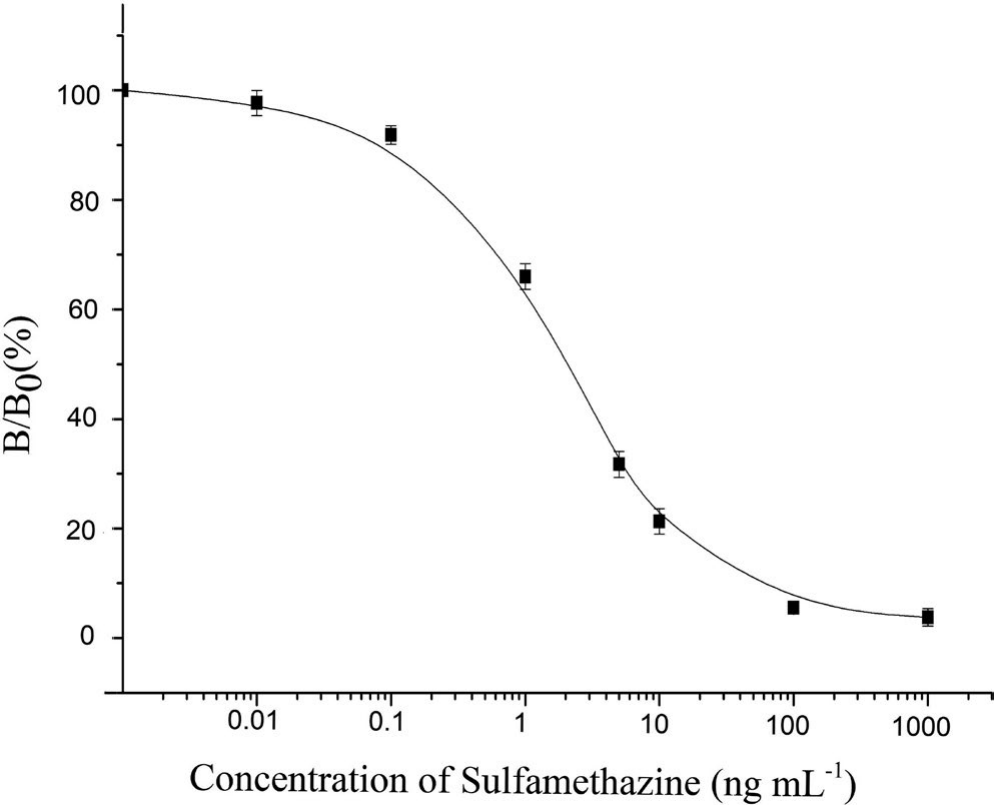
Calibration curves of sulfamethazine. Each point represents the mean of three replicates

Under the optimized conditions, the standard curves of STZ are described in Figure 4, and the LOD and IC_50_ values for STZ were 0.015 ng/ml and 2.11 ng/ml, respectively. In this study, the broad specificity of the SAs AlphaLISA method was evaluated by the cross‐reactivities against 24 SAs. As shown in Table 2, SAs AlphaLISA method showed high sensitivities for 13 SAs including SMT, SMD, SFM, SMR, SMM, SQX, SDM, SDZ, SCP, SCL, SMZ, SXL, and SMP under optimized conditions. The IC_50_ values of the 13 SAs were from 2.11 to 29.77 ng/ml in buffer; the LODs of these SAs ranged from 0.015 to 2.056 ng/ml in buffer. Owing to the real sample being diluted 20‐fold in preparation, the milk and muscle LODs of these SAs ranged from 0.30 to 41.12 ng/ml, which could satisfy the MRL of the European Union, United States, and China.

**TABLE 2:** LOD, IC_50,_ and cross‐reactivities of SAs AlphaLISA method against the 24 selected sulfonamides

SAs	LOD (ng/mL)	IC_50_ (ng/mL)	CR (%)
Sulfamethazine	0.015	2.11	100
Sulfamethoxydiazine	0.066	3.84	55.1
Sulfisomidine	0.078	4.68	45.2
Sulfamerazine	0.094	6.40	33.1
Sulfamonomethoxine	0.095	6.98	30.3
Sulfaquinoxaline	0.112	8.54	24.8
Sulfadimethoxine	0.207	10.74	19.7
Sulfadiazine	0.377	11.95	17.7
Sulfachloropyridazine	0.805	14.78	14.3
Sulfaclozine	0.968	15.94	13.3
Sulfamethizole	1.383	27.81	7.6
Sulfamoxole	1.956	29.39	7.2
Sulfamethoxypyridazine	2.056	29.77	7.1
Sulfadoxine	–	162.79	1.3
Sulfathiazole	–	211.21	1.0
Sulfapyridine	–	323.27	0.7
Sulfamethoxazole	–	475.06	0.4
Sulfisoxazole	–	905.51	0.2
Trimethoprim	–	>1,000	<0.1%
Sulfanitran	–	>1,000	<0.1%
Sulfaguanidine	–	>1,000	<0.1%
Sulfaphenazole	–	>1,000	<0.1%
Sulfacetamide	–	>1,000	<0.1%
Sulfabenzamide	–	>1,000	<0.1%

### 3.4 Validation of the AlphaLISA method

To assess the accuracy and precision of the SAs AlphaLISA method, 3 levels of SMT at 25, 50, and 100 ng/g were added to milk, pork, fish, chicken, and plasma, respectively. As shown in Table 3, the recovery values varied from 87.8% to 116.8%, with the CVs less than 9.3%, which confirmed the AlphaLISA method's effectiveness for SAs detection in different matrices. In addition, these results further support the feasibility of the developed SA AlphaLISA method for subsequent applications in monitoring animal‐derived foodstuffs.

**TABLE 3:** Recoveries of sulfamethazine in 5 matrices using AlphaLISA method

Matrix	Spiked level (ng/g)	Recoveries (%)	CVs (%)
Milk	25	103	2.5
	50	109	9.3
	100	110.7	1.8
Pork	25	88	2.8
	50	116.8	4.9
	100	87.8	5.1
Fish	25	116	6.8
	50	88.2	6
	100	97.1	5.2
Chicken	25	116	6.6
	50	101	5.7
	100	93.2	8.4
Plasma	25	91.2	1.9
	50	83.6	7.9
	100	98.8	4.9

**FIGURE 5:**
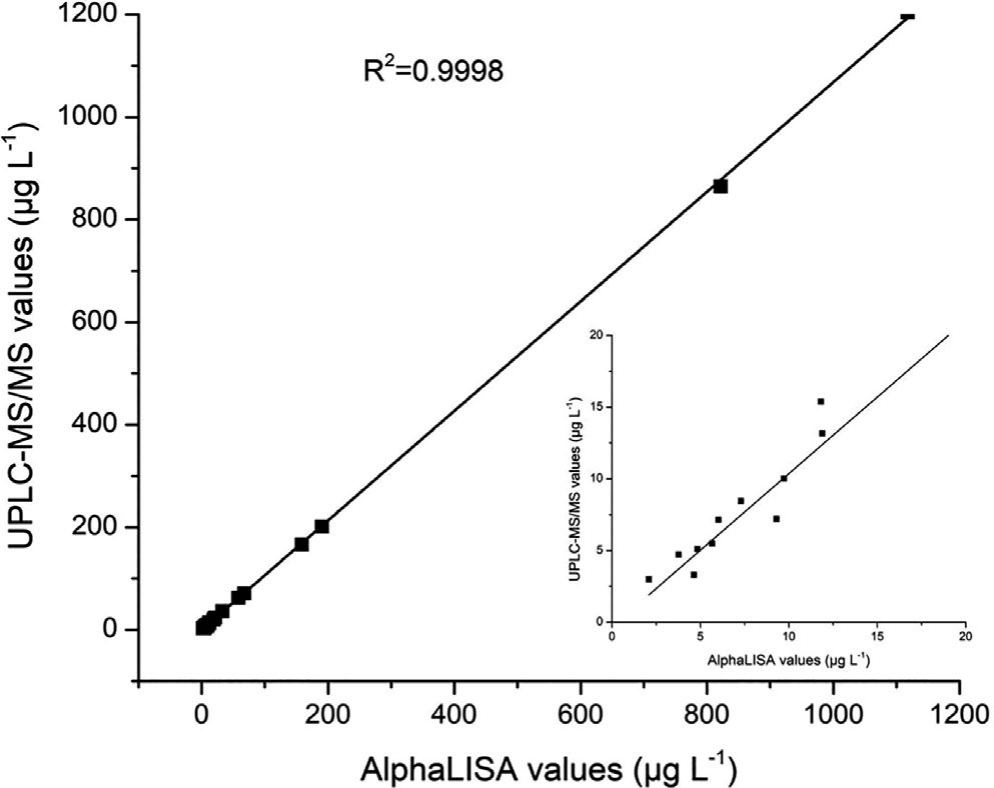
Correlation of sulfamethazine detection in plasma between the UPLC‐MS/MS and AlphaLISA methods. (Inset) Low sulfamethazine concentration range detected by UPLC‐MS/MS and AlphaLISA methods

In order to further evaluate the detection capability and the accuracy of the SAs AlphaLISA method, the rabbit plasmas with SMT were analyzed by the SA AlphaLISA and further validated using UPLC‐MS/MS. As shown in Figure 5, the concentrations of SMT in different samples determined by AlphaLISA method are in good agreement (*R* ^2^ = 0.9998) with those determined by UPLC‐MS/MS. Therefore, this SAs AlphaLISA method is reliable for SAs residue detection in foods of animal origin.

Furthermore, the maximum concentrations of SMT residues were 864.5 and 1,203.5 µg/kg found in the plasma of the two rabbits by AlphaLISA and decreased quickly. At 3 hr postadministration, the concentrations of SMT residues were considerably below 100 µg/kg, the MRL set by the European Union and China for edible tissues.

## 4 CONCLUSIONS

In this study, we proposed a homogeneous immunoassay based on the AlphaLISA method to detect SAs antibiotic residues in plasma, milk, pork, chicken, and fish. The optimized SAs competitive AlphaLISA method, which satisfied the MRL of the European Union, United States, and China, could monitor 13 kinds of SAs in animal‐based foods. The method showed small‐volume samples (15 μL), low limits of detection (in the range of 0.3–41.12 ng/ml in the various matrices), and high recovery rates (87.8%–116.8% in SMT‐dd plasma, milk, pork, chicken, and fish samples). The good correlations between the AlphaLISA and UPLC‐MS/MS for blood samples indicating the AlphaLISA method offer better reproducibility in addition to its straightforward operation. Moreover, the AlphaLISA is almost free from influence of hindrances such as sample with complex matrices for food detection. These advantages of AlphaLISA method fit more for the automatic operations in the future.

## AUTHOR CONTRIBUTIONS

**yong Jin:** Methodology (lead); Writing‐original draft (lead). **yanping He:** Investigation (equal); Methodology (equal); Writing‐original draft (equal). **dali Zhao:** Data curation (equal); Investigation (supporting). **yan Chen:** Investigation (supporting). **qiang Xue:** Methodology (supporting). **Mingqiang Zou:** Project administration (equal). **Hong Yin:** Writing‐review & editing (supporting). **shige xing:** Methodology (equal); Writing‐review & editing (lead).

### 1 CONFLICTS OF INTEREST

No conflict of interest declared.

## ETHICAL APPROVAL

The animal testing involved in this study was reviewed and approved by Institutional Animal Care and Use Committee (IACUC) and was conducted in accordance with relevant guidelines and regulations.
